# A Mendelian randomization study of genetic liability to post-traumatic stress disorder and risk of ischemic stroke

**DOI:** 10.1038/s41398-023-02542-y

**Published:** 2023-07-01

**Authors:** Opeyemi Soremekun, Clarisse Musanabaganwa, Annette Uwineza, Maddalena Ardissino, Skanda Rajasundaram, Agaz H. Wani, Stefan Jansen, Jean Mutabaruka, Eugene Rutembesa, Chisom Soremekun, Cisse Cheickna, Mamadou Wele, Joseph Mugisha, Oyekanmi Nash, Eugene Kinyanda, Dorothea Nitsch, Myriam Fornage, Tinashe Chikowore, Dipender Gill, Derek E. Wildman, Leon Mutesa, Monica Uddin, Segun Fatumo

**Affiliations:** 1 The African Computational Genomics (TACG) Research group, MRC/UVRI and LSHTM, Entebbe, Uganda; 2 Discipline of Pharmaceutical Chemistry, College of Health Sciences, University of KwaZulu-Natal, Durban, South Africa; 3 Medical Research Center, Rwanda Biochemical Centre, Kigali, Rwanda; 4 Department of Biochemistry, Molecular Biology and Genetics, CMHS, University of Rwanda, Kigali, Rwanda; 5 Center for Human Genetics at the College of Medicine and Health Sciences-University of Rwanda, Kigali, Rwanda; 6 Department of Epidemiology and Biostatistics, Medical School Building, St Mary’s Hospital, Imperial College London, London, UK; 7 Centre for Evidence-Based Medicine, University of Oxford, Oxford, UK; 8 Faculty of Medicine, Imperial College London, London, UK; 9 Genomics Program, College of Public Health, University of South Florida, Tampa, FL USA; 10 Directorate of Research and Innovation, University of Rwanda, Kigali, Rwanda; 11 Department of Clinical Psychology, University of Rwanda, Kigali, Rwanda; 12 H3Africa Bioinformatics Network (H3ABioNet) Node, Centre for Genomics Research and Innovation, NABDA/FMST, Abuja, Nigeria; 13 The African Center of Excellence in Bioinformatics, University of Sciences, Techniques and Technologies of Bamako (USTTB), Bamako, Mali; 14 MRC/UVRI and LSHTM, Entebbe, Uganda; 15 Department of Non-communicable Disease Epidemiology (NCDE), London School of Hygiene and Tropical Medicine, London, UK; 16 Brown Foundation Institute of Molecular Medicine, McGovern Medical School, University of Texas Health Science Center at Houston, Austin, USA; 17 Human Genetics Center, School of Public Health, University of Texas Health Science Center at Houston, Austin, USA; 18 MRC/Wits Developmental Pathways for Health Research Unit, Department of Pediatrics, Faculty of Health Sciences, University of the Witwatersrand, Johannesburg, South Africa; 19 Sydney Brenner Institute for Molecular Bioscience, Faculty of Health Sciences, University of the Witwatersrand, Johannesburg, South Africa; 20 Channing Division of Network Medicine, Department of Medicine, Brigham and Women’s Hospital and Harvard Medical School, Boston, USA

**Keywords:** Genomics, Medical genetics

## Abstract

Observational studies have shown an association between post-traumatic stress disorder (PTSD) and ischemic stroke (IS) but given the susceptibility to confounding it is unclear if these associations represent causal effects. Mendelian randomization (MR) facilitates causal inference that is robust to the influence of confounding. Using two sample MR, we investigated the causal effect of genetic liability to PTSD on IS risk. Ancestry-specific genetic instruments of PTSD and four quantitative sub-phenotypes of PTSD, including hyperarousal, avoidance, re-experiencing, and total symptom severity score (PCL-Total) were obtained from the Million Veteran Programme (MVP) using a threshold *P* value (*P)* of <5 × 10^−7^, clumping distance of 1000 kilobase (Mb) and *r*^2^ < 0.01. Genetic association estimates for IS were obtained from the MEGASTROKE consortium (*N*_cases_ = 34,217, *N*_controls_ = 406,111) for European ancestry individuals and from the Consortium of Minority Population Genome-Wide Association Studies of Stroke (COMPASS) (*N*_cases_ = 3734, *N*_controls_ = 18,317) for African ancestry individuals. We used the inverse-variance weighted (IVW) approach as the main analysis and performed MR-Egger and the weighted median methods as pleiotropy-robust sensitivity analyses. In European ancestry individuals, we found evidence of an association between genetic liability to PTSD avoidance, and PCL-Total and increased IS risk (odds ratio (OR)1.04, 95% Confidence Interval (CI) 1.007–1.077, *P* = 0.017 for avoidance and (OR 1.02, 95% CI 1.010–1.040, *P* = 7.6 × 10^−4^ for PCL total). In African ancestry individuals, we found evidence of an association between genetically liability to PCL-Total and reduced IS risk (OR 0.95 (95% CI 0.923–0.991, *P* = 0.01) and hyperarousal (OR 0.83 (95% CI 0.691–0.991, *P* = 0.039) but no association was observed for PTSD case-control, avoidance, or re-experiencing. Similar estimates were obtained with MR sensitivity analyses. Our findings suggest that specific sub-phenotypes of PTSD, such as hyperarousal, avoidance, PCL total, may have a causal effect on people of European and African ancestry’s risk of IS. This shows that the molecular mechanisms behind the relationship between IS and PTSD may be connected to symptoms of hyperarousal and avoidance. To clarify the precise biological mechanisms involved and how they may vary between populations, more research is required.

## Background

Post-Traumatic Stress Disorder (PTSD) has been described as an extreme response to traumatic conditions [1]. Several risk factors are implicated in the aetiology of PTSD, such as exposure to violence [1], childhood trauma [2], and educational attainment [3, 4]. However, not all individuals exposed to traumatic events end up developing PTSD [5]. The Diagnostic and Statistical Manual of Mental Disorders (DSM), DSM-IV [6], and DSM-5 [7] both employ the PTSD Checklist (PCL) to identify the symptoms of PTSD. A revised version of the PCL, known as the PCL-5, was designed to assess PTSD symptoms as defined in the DSM-5 whereas the original PCL was designed to evaluate PTSD symptoms as defined in the DSM-IV. The DSM-5 PTSD symptoms are represented by 20 items in the PCL-5. The symptoms categorization includes Exposure to actual or threatened death, serious injury, or sexual violence. Intrusive and distressing thoughts, memories, or dreams related to the traumatic event(s). Avoidance of people, places, or things that remind the person of the traumatic event(s). Negative changes in mood or cognition, such as persistent negative emotions, distorted thoughts about the event, and feelings of detachment or estrangement from others. Increased arousal or reactivity, including difficulty sleeping, irritability, anger outbursts, and hypervigilance.

The most common type of stroke, ischemic stroke (IS), is caused by a blockage in the circulatory arteries that carry blood to the brain. IS results in ~80% of all adult stroke cases [8]. The 10-year survival rate following an ischemic stroke is 25%, whereas the survival rate following a hemorrhagic stroke is 18% [9].

Observational studies support an association between PTSD and IS risk [10–14]. A growing body of research also indicates that PTSD predicts stroke. For example, in a longitudinal study, individuals with PTSD had a higher risk of any stroke (hazard ratio (HR) 3.37, 95% CI 2.44–4.67 and ischemic stroke (HR = 3.47, 95% CI 2.23–5.39) after controlling for demographic data and medical comorbidities [10]. The biological basis of the relationship between PTSD is poorly understood. However, a few viable explanations have been put forth suggesting possible biological underpinnings of stroke. One hypothesis is that the outcome of stroke can be attributed to the physiological impacts of long-term stress on the body, which can affect the cardiovascular system by altering immunological function, increasing inflammation and oxidative stress, and altering blood vessel response to stress [15, 16].

However, observational studies are inherently limited in estimating causal relationships due to a vulnerability to confounders, amongst a number of other limitations [17–20]. Mendelian randomization (MR) can be used to circumvent this challenge and to date has not been leveraged to investigate this question. MR utilises genetic variants in an instrumental variable framework to investigate the causal relationship between a putative biological risk factor and a disease of interest [21, 22].

We therefore, used MR to investigate the relationship between genetic liability to PTSD and PTSD sub-phenotypes—hyperarousal, avoidance, re-experiencing, and PCL-Total (summation of the three quantitative phenotypes) as defined by Stein et al. [23], with risk of IS in European and African ancestry individuals.

## Methods

### Study populations

Genome-wide association study summary statistics for PTSD traits were obtained from the Million Veteran Program (MVP) for both African and European ancestry individuals (Supplementary ST1). The MVP comprises five PTSD phenotype consisting of a case-control phenotype and four quantitative PTSD phenotypes: re-experiencing, avoidance, hyperarousal, and total index of recent symptom severity (Table 1) [23]. The MVP obtained ethical approval from the Veteran Affairs Central Institutional Review Board in accordance with the principles outlined in the Declaration of Helsinki. Genetic association estimates for IS risk in African ancestry individuals were obtained from the Consortium of Minority Population Genome-Wide Association Studies of Stroke (COMPASS). COMPASS is a GWAS meta-analysis of 13 African ancestry cohorts including 3734 cases and 18,317 controls (Supplementary ST1). All participants provided written, informed consent, and institutional review boards approved each of the respective studies [24]. Genetic association estimates for IS and other stroke subtypes in European ancestry individuals were obtained from the MEGASTROKE consortium (34,217 cases and 406,111 controls) [25].

**Table 1:** Description of the sample size and strength of instrumental variables.

Ancestry	PTSD phenotype	Sample size	No. SNPs
AFRICANS	Case-control	*N* cases = 11,920, *N* control = 39,116	4
	Hyperarousal	25,521	2
	Avoidance	25,414	9
	Re-experiencing	25,414	3
	PCL total	25,318	3
EUROPEANS	Cas-control	*N* cases = 36,301, *N* control = 178,107	5
	Hyperarousal	186,689	33
	Avoidance	186,689	30
	Re-experiencing	186,689	26
	PCL total	186,689	35

### Statistical analyses

We constructed our genetic instrument for each PTSD trait in three steps. First, SNPs reaching a threshold of *P* < 5 × 10^−7^ were selected from the corresponding PTSD trait GWAS. This threshold was used because in several PTSD traits a portion of SNPs did not reach the standard genome-wide significance threshold of *P* < 5 × 10^−8^. To therefore increase the number of SNPs available for the primary and secondary analyses, we used *P* < 5 × 10^−7^. Secondly, we pruned these SNPs and selected independent SNPs by using a clumping window of 1000 kb and *r*^2^ < 0.01. Genetic association estimates for these SNPs were extracted from COMPASS and MEGASTROKE summary statistics for African and European ancestry, respectively. Third, we harmonised the effects by ensuring that the effect of the SNP on the exposure and the effect of the SNP on the outcome had the same effect allele. Palindromic SNPs with effect allele greater than 40% were not considered as viable instruments. In our current analysis, we made the necessary assumptions for MR analysis, i.e., instrumental variables (IVs) must be strongly associated with exposure of interest, and the IVs only influence the outcome phenotype via effects on risk factors [20, 26]. Any SNP that violated these assumptions was considered an invalid IV because it may cause bias in MR analysis. We used inverse-variance weighted (IVW) as the main MR method in this research. The causal estimate β was calculated with the equation *w*_i_ (*α*_i_/*γ*_i_). i refers to the IVs, α_i_ connotes the association effect of IVs on IS, γ_i_ signifies the association effect of IVs with PTSD, and w_i_ the weights of the causal effect of PTSD on IS. Where we have less than two instruments, Wald ratio was used as the main test. To corroborate the results of the MR-IVW estimates, we used MR-Egger and the weighted median MR method to determine the association of PTSD genetic liability with IS. MR-Egger accommodates the estimation of an intercept, a statistically significant intercept indicates the presence of unbalanced pleiotropy which subsequently suggests that the IVW MR estimates are more precise than the MR-Egger estimate [21]. Weighted median MR can compute causal estimates even if 50% IVs are invalid [22]. The weighted median method employs inverse-variance weight and bootstrapping in the estimations of confidence interval [22]. To estimate the strength of our instruments, we computed *F*- statistics for each SNP using method described by Burgess et al. [27].

## Results

### Genetic liability to PTSD phenotypes and stroke risk

**Fig. 1:**
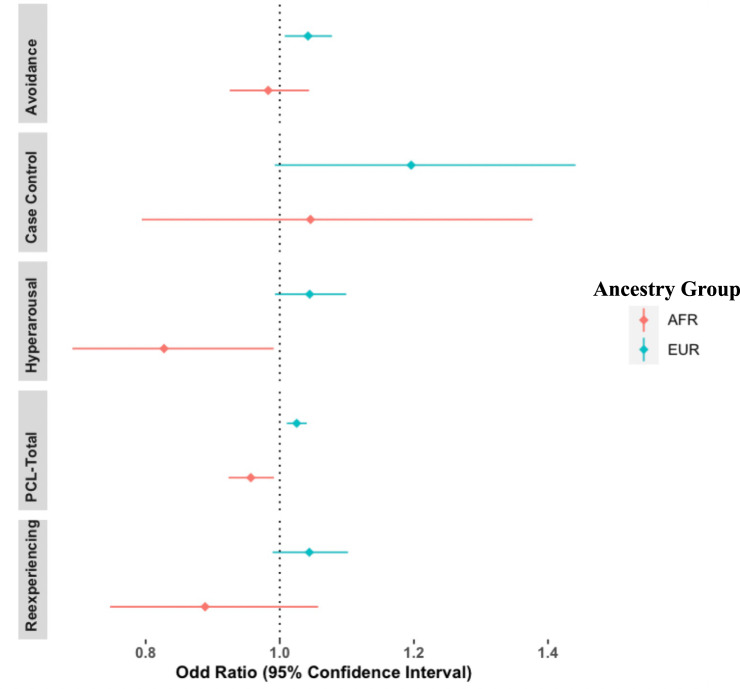
Forest plot summarizing the effect of genetically predicted PTSD traits on IS risk in African and European populations.

In African ancestry analysis the *F*-statistics of all the instrumental variables used were >10. Detailed information on the instruments is in supplementary ST2–ST6. We found no MR evidence of association between genetic liability to PTSD case-control, avoidance, re-experiencing, and IS. There was however evidence of association of genetic liability to PTSD PCL-Total and hyperarousal with decreased risk of IS. The OR of genetically predicted PTSD case-control against IS in African ancestry individuals was 1.05 (95% CI 0.794, 1.377; *P* = 0.750), PTSD hyperarousal against IS 0.83 (95% CI 0.691, 0.991; *P* = 0.039), PTSD avoidance against IS 0.98 (95% CI 0.925, 1.044; *P* = 0.569), PTSD re-experiencing against IS 0.88 (95% CI 0.747, 1.057; *P* = 0.182), and PTSD PCL-Total against IS 0.95 (95% CI 0.923, 0.991; *P* = 0.014) (Fig. 1, supplementary ST7–ST11).

In the European ancestry analysis five, 33, 30, 26, and 35 instrumental variables from PTSD case-control, hyperarousal, avoidance, re-experiencing, and PCL-Total respectively were selected from the MVP summary statistics (supplementary ST12–ST16). We found evidence of the association between genetic liability to PTSD avoidance, and PCL-Total with increased risk of IS. PTSD case-control, hyperarousal, and re-experiencing showed no statistical association with IS. The odds ratio (OR) of the genetically predicted exposure with IS [OR] per 1-standard deviation increase in PTSD traits were 1.19, 95% CI 0.992–1.441, *P* = 0.060 (PTSD case-control), 1.04, 95% CI 0.992–1.098, *P* = 0.093 (hyperarousal), 1.04, 95% CI 1.007–1.078, *P* = 0.017 (avoidance), 1.04, 95% CI 0.989–1.102, *P* = 0.117 (re-experiencing), and 1.02, 95% CI 1.010–1.040, *P* = 7.6 × 10^−4^ (PCL-Total) (Fig. 1, Supplementary ST17–ST21).

For sensitivity analysis, we used three different methods (simple median-based, weighted median-based, and MR-Egger). We did not detect any substantial horizontal pleiotropy in our analyses as indicated by the MR-Egger intercept *P* value (intercept close to zero and *P* > 0.05). The sensitivity analyses showed consistent results indicating robustness of the MR-Egger findings (Supplementary ST7–ST11, ST17–ST21). Similarly, no evidence of heterogeneity was observed (supplementary ST22) as evidenced by the non-significance of the test statistics (supplementary ST22) using MR-PRESSO. A detailed pleiotropy description of the genetic instruments is shown in supplementary ST22. In the reverse analysis, we found no evidence of significant causal relationship between IS liability against PTSD traits in either ancestral group (Supplementary ST23–ST42).

## Discussion

### Statement of findings

This MR study investigated the relationship between genetic liability to PTSD traits and IS risk in European and African ancestry individuals. In European ancestry individuals, we found evidence of an association between genetic liability to PTSD phenotypes including hyperarousal, avoidance, and PCL-Total, and an increased risk of IS. In evaluating the relationship between PTSD traits and the risk of other Stroke subtypes (LAS, CES, and SVS), we found no evidence of MR association between PTSD traits and stroke subtypes, except between PCL-Total with CES and between avoidance and SVS. This finding was further corroborated in pleiotropy-robust sensitivity analyses. In African ancestry individuals, no such association was found between the four PTSD phenotypes (case-control, avoidance, re-experiencing, and hyperarousal) and IS risk. However, PCL-Total was genetically associated with a reduced risk of IS in African ancestry individuals.

Our MR findings in European ancestry individuals are consistent with several observational studies that have reported that PTSD is a risk factor for IS [10, 28–33]. Several observational studies have found that PTSD is associated with higher stroke risk while adjusting for selected stroke risk factors [10, 28–33]. For example, after adjusting for depression, hypertension, dyslipidemia, and diabetes, a prospective study from the Taiwan National Health Insurance Research Database supported a threefold increase in IS in 5217 individuals with PTSD [34]. In another study, PTSD was shown to be associated with an increased risk of IS and transient ischemic attacks, the association remained consistent after further adjusting other stroke risk factors [28].

Furthermore, although no prior MR studies have explored the association between PTSD traits and IS risk, the genetic association between genetically predicted liability to depression and stroke risk has been previously investigated [35, 36] Using an inverse-variance weighted method, Gill et al., found no evidence of genetically determined risk of depression on IS risk in European population [35]. Similarly, Cai et al. found no association between major depressive disorder and large artery stroke, cardioembolic stroke, and/or IS [36].

Several factors may explain the observed differences in the genetic associations between PTSD traits and IS risk in European versus African ancestry individuals. First, the prevalence and expression of different PTSD traits may differ between European and African ancestry individuals and thus the proportion of IS risk that is attributable to PTSD traits may differ between the two ethnicities [37–40]. For example, although Mainous et al. found no differences in symptoms severity between Europeans and African ancestry individuals, after adjusting for trauma type, Europeans reported more anguish from re-experiencing, hyperarousal, and avoidance symptoms in response to nonphysical trauma than African ancestry individuals [41]. Second, there may be ethnic differences in questionnaire response style, which is defined as an individual’s manner of answering questions such as psychological questionnaires [42]. These cultural differences are worth noting because they have the potential to bias PTSD diagnosis or may amplify or shroud the severity of the trauma. In cross-cultural situations, Western psychiatric diagnostic instruments for PTSD diagnosis may fall short of reflecting the full range of trauma reactions [43]. Instead, questionnaires that have undergone cross-cultural harmonization should be used to capture significant ancestral differences in individuals. Third, due to the dense linkage disequilibrium block within the African genome, it is possible the underlying interaction between different genetic and epigenetic differs in European versus African ancestry individuals.

The mechanisms underlying the effect of PTSD on IS may arise through behaviors such as smoking [44, 45], low exercise [45, 46], poor sleep [47, 48], and poor diet [47]. Another possible explanation is that lengthened exposure to psychological stress is connected to endothelial dysfunction, inflammation, platelet activation, and autonomic dysregulation, all of which have been shown to promote atherothrombotic incidents [45, 49–51].

### Strengths

One major strength of our analysis is the reduced susceptibility of MR to confounding in comparison to traditional epidemiological studies. In our secondary analysis, we used different pleiotropy-robust MR methods that make different analytic assumptions, hence strengthening the credibility of this study. The data used in our analysis for both exposure and outcome phenotypes were strictly from African ancestries individuals, hence, there were no population biases in our analysis. Furthermore, there was no sample overlap between the exposure and outcome datasets.

### Limitations

One limitation of this study is the number of instruments used in the African ancestry analysis, which were fewer (*n* = 21) than those used in the European ancestry analysis (*n* = 129). Rather than biological differences in the relationship between PTSD and stroke across ethnicities, it is possible that the comparatively smaller sample size of African ancestry individuals and subsequent lack of statistical power may partly explain differences between European and African ancestry individuals. We used a *P* value (*P)* of < 5 × 10^−7^ to select SNPs for our genetic instruments, this may have resulted in the inclusion of weak or invalid instruments, however, this was compensated by the *F*-statistics of >10. Other potential drawbacks of this study are that characteristic of MR analysis and assumptions [52]. As discussed above, another potential limitation is misclassification of PTSD across populations, especially in populations where scores are less validated, such as in African populations. This has necessitated a need to ensure continuous advocacy for people to report symptoms of PTSD. The MVP data is predominantly male while the COMPASS data is predominantly female, this is a potential limitation as it is known that sex and gender differences play roles in disease heterogeneity.

## Conclusion

Our study showed that there may be differences in the genetic susceptibility to the development of PTSD and its associated risks for ischemic stroke between individuals of European and African ancestries. This highlights the importance of considering genetic and ancestral diversity when investigating the genetic basis of complex disorders such as PTSD and its related outcomes.

## Supplementary information

Supplementary Tables

## Data Availability

All data used in this study are publicly available unless otherwise stated.
